# Phase I clinical trial of the vaccination for the patients with metastatic melanoma using gp100-derived epitope peptide restricted to HLA-A*2402

**DOI:** 10.1186/1479-5876-8-84

**Published:** 2010-09-16

**Authors:** Toshiyuki Baba, Marimo Sato-Matsushita, Akira Kanamoto, Akihiko Itoh, Naoki Oyaizu, Yusuke Inoue, Yutaka Kawakami, Hideaki Tahara

**Affiliations:** 1 Department of Surgery and Bioengineering, Advanced Clinical Research Center, Institute of Medical Science, The University of Tokyo, 4-6-1 Shirokane-dai, Minato-city, Tokyo, 108-8639, Japan; 2 Department of Pathology and Laboratory Medicine, Institute of Medical Science, The University of Tokyo, 4-6-1 Shirokane-dai, Minato-city, Tokyo, 108-8639, Japan; 3 Department of Radiology of Research Hospital, Institute of Medical Science, The University of Tokyo, 4-6-1 Shirokane-dai, Minato-city, Tokyo, 108-8639, Japan; 4 Division of Cellular Signaling, Institute for Advanced Medical Research, Keio University, School of Medicine, 35 Shinano-machi, Shinjuku-city, Tokyo 160-8582, Japan

## Abstract

**Background:**

The tumor associated antigen (TAA) gp100 was one of the first identified and has been used in clinical trials to treat melanoma patients. However, the gp100 epitope peptide restricted to HLA-A*2402 has not been extensively examined clinically due to the ethnic variations. Since it is the most common HLA Class I allele in the Japanese population, we performed a phase I clinical trial of cancer vaccination using the HLA-A*2402 gp100 peptide to treat patients with metastatic melanoma.

**Methods:**

The phase I clinical protocol to test a HLA-A*2402 gp100 peptide-based cancer vaccine was designed to evaluate safety as the primary endpoint and was approved by The University of Tokyo Institutional Review Board. Information related to the immunologic and antitumor responses were also collected as secondary endpoints. Patients that were HLA-A*2402 positive with stage IV melanoma were enrolled according to the criteria set by the protocol and immunized with a vaccine consisting of epitope peptide (VYFFLPDHL, gp100-in4) emulsified with incomplete Freund's adjuvant (IFA) for the total of 4 times with two week intervals. Prior to each vaccination, peripheral blood mononuclear cells (PBMCs) were separated from the blood and stored at -80°C. The stored PBMCs were thawed and examined for the frequency of the peptide specific T lymphocytes by IFN-γ- ELISPOT and MHC-Dextramer assays.

**Results:**

No related adverse events greater than grade I were observed in the six patients enrolled in this study. No clinical responses were observed in the enrolled patients although vitiligo was observed after the vaccination in two patients. Promotion of peptide specific immune responses was observed in four patients with ELISPOT assay. Furthermore, a significant increase of CD8^+ ^gp100-in4^+ ^CTLs was observed in all patients using the MHC-Dextramer assay. Cytotoxic T lymphocytes (CTLs) clones specific to gp100-in4 were successfully established from the PBMC of some patients and these CTL clones were capable of lysing the melanoma cell line, 888 mel, which endogenously expresses HLA-restricted gp100-in4.

**Conclusion:**

Our results suggest this HLA-restricted gp100-in4 peptide vaccination protocol was well-tolerated and can induce antigen-specific T-cell responses in multiple patients. Although no objective anti-tumor effects were observed, the effectiveness of this approach can be enhanced with the appropriate modifications.

## Background

Multiple tumor associated antigens (TAAs) have been identified and examined for their immunogenicity in clinical trials. The TAAs can be classified into three major categories: cancer/testis (CT) antigens, mutated-gene antigens, and differentiation antigens. The CT antigens are expressed by a large variety of tumors and more than 40 of them have been identified, including MAGE [1], BAGE [2], GAGE [3], XAGE [4], and NY-ESO-1 [5]. Mutated-gene antigens are uniquely present on individual tumors and are rarely shared by many patients. This type of TAA includes β-catenin [6], MUM-1 [7], and CDK-4 [8]. Differentiation antigens are expressed as molecules related to the cell differentiation and have been found mainly in melanomas. These TAAs include MART-1/MelanA [9,10], tyrosinase [11], TRP-1(gp75) [12], and gp100/pMEL 17 [13,14].

The gp100 TAA is a melanocyte lineage-specific membrane glycoprotein consisting of 661 amino acids, categorized as a differentiation Ag. It is expressed in melanomas, but not in other tumor cell types or normal cells with the exception of melanocytes and pigmented cells in the retina. gp100 is recognized by antibodies NKI-beteb, HMB-50 and HMB-45, which are used as diagnostic markers for human melanoma [15]. The reactivity of HMB-45 on formalin-fixed-embedded specimens of malignant melanomas was shown to be approximately 74-80% in large scale studies [16,17]. Thus, gp100 is expressed in most malignant melanomas.

Since HLA-A*0201 is prevalent in Caucasian population, epitope peptides restricted to such allele, gp100:209-217 (ITQVPFSV) [18], and its modified form, gp100:209-217(210M) (IMQVPFSV) which has been modified to have increased binding affinity for HLA-A*0201, have been examined for their immunogenicity [19]. These studies have been shown that these peptides can induce cytotoxic T lymphocytes (CTLs) that recognize cells pulsed with native gp100:209-217 peptide as well as the melanoma cells positive for HLA-A*0201 and gp100 [19]. In other clinical trials, HLA-A*0201-positive melanoma patients were vaccinated with gp100:209-217(210M) with incomplete fluid adjuvant (IFA). In 10 of 11 patients vaccinated with this peptide there was a significant increase in antigen-specific CTL-precursors [20]. Furthermore, 13 of 31 patients treated with gp100:209-217(210M) along with systemic administration of high-dose IL-2 exhibited an objective cancer response. Of these HLA-A*0201 restricted epitope peptides derived from gp100, there are several reports describing successful induction of anti-tumor CTLs in a class I-restricted fashion [21,22]. Thus, epitope peptides derived from gp100 appear to be promising Ags for tumor-specific immunotherapy against malignant melanoma.

In contrast to these HLA-A*0201-restricted peptides, the gp100-derived epitope peptides restricted to HLA-A*2402, which is the most common HLA class I allele in the Japanese population [23,24], have not been examined extensively. However, it has been shown that melanoma-reactive CTLs established from the tumor-infiltrating lymphocytes (TILs) of HLA-A*2402-positive patients recognize a non-mutated peptide, encoded by an aberrant transcript of the gp100 gene [25]. This transcript contains the fourth intron of the gp100 gene and the CTL epitope is encoded within this region. The peptide, termed gp100-in4 (VYFFLPDHL), was observed to be expressed only at low levels, but the CTLs can recognize very small amounts of the cell surface HLA/peptide complex. In addition, gp100-in4 binds to HLA-A*2402 with high affinity and thus might be very efficiently processed and present on the melanoma and melanocyte cell surface. The binding affinity of gp100-in4 was predicted to be very high at the score of 240.0, when the analysis was performed with the computer-based program for molecular analysis section (BIMAS) for HLA peptide binding predictions [26]. Thus, gp100-in4 might be the most promising epitope peptide among the candidate peptides derived from gp100 to treat HLA-A*2402-positive melanoma patients.

We have conducted a phase I clinical trial to treat the HLA-A*2402-positive patients with stage IV melanoma by vaccination with the gp100-in4 peptide. In this study, we examined the safety of this treatment as a primary endpoint and the clinical and immunological responses as secondary endpoints. For the immunological monitoring, we employed both ELISPOT and MHC-Dextramer assays. Furthermore, CTL clones specific to gp100-in4 were established from peripheral blood mononuclear cells (PBMCs) of the treated patients and analyzed for their functions.

## Methods

### Patients

All patients were diagnosed to have stage-IV melanoma based on the American Joint Commission on Cancer staging system and had received various treatments prior to the entry of this protocol. These treatments include surgery, chemotherapy, radiation therapy, thermotherapy and immunotherapy, all of which have failed prior to enrollment. Other eligibility criteria included the age (20-75 years), HLA typing (HLA-A*2402), existence of tumor lesions measurable with CT or MRI, good performance status (0 to 2 in the Criteria of Eastern Cooperative Oncology Group (ECOG), adequate bone marrow, hepatic, and renal functions (WBC >3000/mm^3 ^and <9000/mm^3^; AST <50 U/ml; ALT <50 U/ml; serum bilirubin <1.1 mg/dl; BUN <20 mg/dl; and creatinine <1.1 mg/dl). Patients also were required to receive no treatment for the disease for four weeks prior to the initiation of the vaccination and have no serious infection. Exclusion criteria included having less than 3 months of expected survival and receiving corticosteroids or immunosuppressive drugs. Women who were pregnant and lactating also were not eligible. All patients were gave written informed consent to participate in the study according to the Declaration of Helsinki and the study was approved by the University of Tokyo Institute of Medical Science IRB. Tumor responses to the treatment were assessed according to Response evaluation criteria in solid tumors (RECIST).

### Study design and treatments

The study was an open-label phase I study in patients with advanced malignant melanoma to assess safety of the treatment. Immunological response and clinical responses were examined as secondary endpoints. The enrolled patients were treated with a vaccine composed of the gp100-derived epitope peptide restricted to HLA-A*2402 every two weeks for four times in total as one course. Additional courses of the treatments were allowed after having the approval of the case management committee. The study protocol was approved by Institutional Review Board (IRB) of the Institute of Medical Science at the University of Tokyo and written informed consent was obtained from all of the patients at the time of enrollment.

### Peptides

The gp100-in4 (VYFFLPDHL) [27], HLA-A*2402-resticted epitope peptide derived from gp100, were used to vaccinate HLA-A*2402 melanoma patients. The peptide was purchased from Multiple Peptide Systems (San Diego, CA) where it was synthesized under current good manufacturing practice conditions defined by US-FDA. For *in vitro *immunological monitoring, the HLA-A*2402-restricted-CMV peptide (QYDPVAALF) was used as a positive control and the HLA-A*2402-restricted-HIV peptide (RYLRDQQLL) [27] was used as a negative control. The HIV and CMV peptides were synthesized by Dr. Shinobu Ohmi (Division of Cell Biology and Biochemistry, Department of Basic Medical Science, Institute of Medical Science, The University of Tokyo).

### Vaccination

The enrolled patients were injected subcutaneously with 1 mg of the gp100-in4 emulsified in 1 ml of IFA (Montanide ISA-51, Seppic, France) into the skin at the axillary or inguinal region. Four vaccinations with gp100-in4 were given at two week intervals in one course. A physical examination, blood cell counts and standard blood tests were performed prior to each vaccination and 2 weeks after the last vaccination.

### Peripheral blood samples

The 50 ml of peripheral blood was drawn from the enrolled patients before each vaccination and 2 weeks after the last vaccination for the immunological monitoring. Drawn blood was heparinized, prepared for PBMCs with Ficoll-Paque (Amersham Biosciences, Piscataway, NJ) gradient centrifugation, and suspended in heat-inactivated human AB serum (MP Biomedicals, Irvine, CA) with 10% DMSO (Wako Pure Chemical industries, Ltd., Osaka, Japan), for cryopreservation in a liquid nitrogen freezer at -180°C.

### Cell lines

The A24-LCL, a human B-lymphoblastoid cell line (B-LCL) which expresses the HLA-A24 allele, was pulsed with peptides and used for a stimulator or target in cytotoxicity assay. An Epstein-Barr virus-transformed B-lymphoblastoid cell line, EHM (HLA-A03/03), was used for the expansion of CTLs. These cell lines were cultured in RPMI1640 medium (GIBCO, Grand Island, NY) containing 100 U/ml of penicillin, 100 mg/ml of streptomycin (GIBCO), and 10% heat-inactivated fetal bovine serum (FBS) (Sigma Diagnostics, St. Louis, MO).

A colon adenocarcinoma cell line, HT29 (HLA-A1/A24) purchased from American Type Culture Collection (ATCC; Manassas, VA), and melanoma cell lines, 888 mel (HLA-A1/A24) and 397 mel (HLA-A1/A25) established [28], were maintained in Dulbecco's modified Eagle's medium (GIBCO) supplemented with 100 U/ml of penicillin, 100 mg/ml of streptomycin, and 10% heat-inactivated FBS. They were used as targets to examine the cytotoxicity of CTLs raised from the patients' PBMC.

### Cell culture for immunological assays

The cryopreserved PBMCs of the patients were thawed and suspended at the density of 1 × 10^7 ^cells per 15 ml tube (Falcon) in 10 ml with RPMI1640 medium supplemented with 10% FBS, 100 mg/ml streptomycin, 100 IU/ml penicillin, and 5 × 10^-5 ^M 2-mercaptoethanol (all from Invitrogen Life Technologies), referred to henceforth as complete medium. After rinsing the cells twice with the complete medium, 1 × 10^6 ^of PBMCs were cultured in the 2 ml of complete medium supplemented with recombinant human (rh) IL-2 (20 U/ml) containing 10 μg/ml of either the gp100-in4 peptide, HIV peptide (a negative control), or CMV peptide (a positive control) using 5 ml round bottom tubes (BD). To ensure better sensitivity and specificity for each assay, ELISPOT and the MHC-Dextramer assays were performed after 4 and 8 days of culture.

### Cytokine-specific Enzyme-Linked Immuno-spot assay

The patients' PBMCs were cultured for 4 days as described above and the IFN-γ-producing cells detected using 96-well nitrocellulose base plates of ELISPOT assay kits (BD™ ELISPOT Research Products, BD Pharmingen) according to the manufacturer's instructions. Briefly, cultured cells were placed at a density of 5 × 10^4 ^cells per well in complete medium with 10 μg/ml of either the gp100-in4, HIV (a negative control), or CMV (a positive control) peptide in pre-coated BD™ ELISPOT plates for 48 hours under conditions of 37°C and 7.5% CO_2_. Spots were developed using biotinylated detection antibody for anti-IFN-γ antibody, streptavidin-HRP, AEC substrate solution. Frequencies of antigen-specific spot-forming cells were measured with C. T. L. Immunospot analyzer and software (Cellular Technologies, Cleveland, OH). Every experiment was performed in quadruplicate.

### MHC-Dextramer analysis

The MHC-Dextramers holding epitope peptides of gp100 (gp100-in4) or HIV (a negative control) were synthesized by Dako Japan Inc. (Tokyo, Japan) and used according to the instruction. 1 × 10^6 ^cells were cultured for 8 days as described above and stained with 10 μl of PE-conjugated MHC-Dextramers for 20 min in the dark at room temperature. All samples then were incubated with 7AAD, APC-conjugated anti-CD3 mAb and FITC-conjugated anti-CD8 mAb in the condition recommended by the manufacturer (BD Pharmingen) for 30 min at 4°C in the dark. Flow cytometric measurements were performed using a FACS Calibur (BD Bioscience) and analyzed using BD CellQuest Pro (BD Biosciences).

### Establishment of CTL clones

CTL clones were generated following a method described previously with minor modifications [29]. Patients' PBMCs were stimulated in culture with the gp100-in4 peptide, and the cells capable of producing IFN-γ at the levels higher than those with HIV peptide (a negative control) were selected and plated in 96-well round bottom plates at 0.3, 1, and 3 cells per well with 8 × 10^4 ^γ-irradiated (3.3 Gy) allogenic PBMCs and 1 × 10^4 ^γ-irradiated (8 Gy) EHM in 150 μl of AIM-V medium containing 5% heat-inactivated human AB serum, 30 ng/ml of anti-CD3 MAb (PharMingen), and 125 IU/ml of rhIL-2 (Teceleukin, Biogen, Inc. Cambridge, MA). Ten days after the stimulation, 50 μl of culture medium containing 500 IU/ml of rhIL-2 was added to each well of the culture. On day 14, the cytotoxic activity of each culture was tested against A24-LCL cells pulsed with either the corresponding peptide or HIV peptide using a ^51^Cr release assay. The CTLs with peptide-specific cytotoxic activities were expanded to characterize their functions in detail [30-32]. The 5 × 10^4 ^of selected CTLs were suspended in 25 ml of AIM-V medium containing 5% heat-inactivated human AB serum and 30 ng/ml of anti-CD3 mAb and co-cultured with 2.5 × 10^7 ^γ-irradiated allogenic PBMCs and 5 × 10^6 ^γ-irradiated EHM in a 25 cm^2 ^Flask (BD Biosciences). One day post-initiation of the culture, 120 IU/ml of rhIL-2 was added to the well. The cultures were supplemented with fresh AIM-V medium containing 5% heat-inactivated human AB serum and 30 IU/ml of rhIL-2 on days 5, 8, and 11. On average, approximately 1-2 × 10^7 ^cells were established as CTL clones by day 14 of the culture.

### Cytotoxicity assays

Cytotoxicity was measured using a standard 4-h ^51^Cr-release assay. The A24-LCL cells were pulsed with 20 μg/ml of either the corresponding peptide or HIV peptide in 10 ml of AIM-V medium overnight and used as targets in cytotoxicity assays. The peptide-pulsed A24-LCL cells and the cancer cell lines (888 mel, 397 mel, and HT29) were labeled with 100 μCi of ^51^Cr for 1 hour at 37°C. Labeled target cells (1 × 10^4 ^in 100 μl/well) were placed into u-bottom-type 96-well micro-culture plates, and the CTL clones, in 100 μ of media, were added to each well as effecter cells to achieve the E/T ratios indicated in the figures. Each assay was performed in duplicate. The supernatants were harvested after 4 hours of incubation, and radioactivity was measured with a γ-counter. Percent specific lysis was calculated as follows: % specific lysis = [(experimental lysis - minimal lysis)/(maximal release - minimal release)] ×100. Minimal lysis was obtained by incubating the target cells with the culture medium alone, and maximal lysis was obtained by exposing the target cells to 1N HCl. In some cytotoxicity assays, target or effecter cells were incubated with blocking Abs as pre-treatments to examine the characteristics of CTLs as described elsewhere [30-32]. These pre-treatments include the incubation of ^51^Cr-labeled 888 mel tumor cells or CTLs with anti-HLA class I mAb, anti-HLA Class II mAb, IgG1, or IgG2a, or with anti-CD4 mAb, anti-CD8 mAb, or IgG1 for 30 min at 4°C, respectively. Then, pre-treated effectors and targets were mixed at an E/T ratio of 30 and cytotoxicity examined as described above. Percent inhibition was determined using the following formula: [(% specific lysis of inhibition by isotype control) - (% specific lysis of inhibition by MAb)]/(% specific lysis of inhibition by isotype control) × 100. All mAb was purchased from Dako Japan Inc.

### Immuno-histochemical analysis

Biopsy specimens were taken from some of vaccinated patients with written informed consent. Serial sections of paraffin-embedded tissues were made and stained with H&E, S100 (Polyclonal rabbit anti-S100, Dako Japan Inc), or monoclonal antibodies against CD3, CD4 (Novocastra Laboratories Ltd, Newcastle upon Tyne, UK), and CD8 (Dako) according to the manufacturers' instructions.

## Results

### Patient characteristics

The characteristics of the HLA-A*2402-positive patients with stage IV melanoma enrolled in this trial (P1-P6) are shown in Table 1. All patients had a score of 0-1 in the performance status scale defined by ECOG. All patients had received other therapies including surgery, chemotherapy, and radiation therapy prior to the enrollment. Four male and two female patients with a median age of 55 (range, 35-74 years) were enrolled.

**Table 1:** Clinical profiles of enrolled patients

Patients	Age	Sex	Primary sites	Sites of metastases	Stage	PS	Previous Tx
P1	41	female	Skin (back)	Liver, Spleen, Skin	IV	0	S/C
P2	74	female	Skin (knee)	Lymph nodes	IV	0	S/C
P3	58	male	Ocular	Liver, Stomach	IV	0	S/C
P4	69	male	Skin (chest)	Lung, Liver	IV	1	S/C
P5	35	male	Ocular	Liver	IV	0	R/C
P6	52	male	Nasal cavity	Lymph node, Skin	IV	0	R

The six HLA-A*2402-positive patients with stage IV melanoma initially enrolled in this clinical trial are shown in this table. All patients had relatively good performance status (PS) and had previously undergone treatments (Tx), for example surgery (S), chemotherapy (C), and radiation therapy (R).

### Adverse events

**Figure 1:**
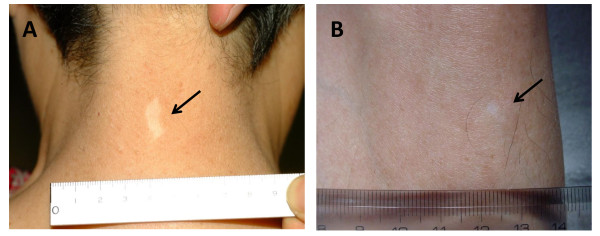
**Vitiligo in patient P2 and P3**. Vitiligo appeared throughout the body during the 1st course of vaccination in P2 and P3. Representative finding of vitiligo was observed in the posterior portion of the neck in P2 (A) and the anterior tibial portion of the left leg in patient P3 (B).

The adverse events observed in all the patients enrolled in this trial are listed in Table 2. Grade III non-hematological adverse events were observed in P1 (CNS hemorrhage at the brain metastasis) and P4 (hypoxia), and grade III hematological adverse events were observed in P2 (anemia). However, all these events were judged to be not related to the treatment, but due to the progress of the disease. Transient dermatologic toxicities such as induration, rubor, local pain, and itching were observed in all patients at the injection sites (grade I toxicity). P2 (Fig.1A) and P3 (Fig.1B) developed vitiligo during the 1st course of vaccination. These results suggest that gp100 peptide-based vaccines was well-tolerated by the enrolled patients.

**Table 2:** Clinical observations on enrolled patients

Patients	Times of vaccination (course)	Follow-up	Adverse events	Clinical anti-tumor responses		Vitiligo
				After 1 course	Final	
P1	12 (3)	13 months	Induration, Rubor, Itching, CNS hemorrhage*	PD	PD	None
P2	8 (2)	8 months	Induration, Rubor, vitiligo, Anemia*	PD	PD	+
P3	8 (2)	7 months	Induration, Rubor, Local pain, vitiligo	PD	PD	+
P4	4 (1)	2 months	Induration, Hypoxia*	PD	PD	None
P5	2 (1)	1 month	Rubor, Pyrexia	incomplete	( - )	None
P6	4 (1)	4 months	Rubor	PD	PD	None

The results of this clinical trial for six patients individually are summarized in this table. P1 showed evidence of an anti-tumor effect. Vitiligo appeared in two patients (P2 and P3) throughout the body. Both anti-tumor effect and vitiligo were defined as the clinical and immunological responses.

### Clinical anti-tumor responses

Table 2 shows the summary of the clinical observations of the enrolled patients. Two patients received one course of vaccination and three received two or more courses. P5 withdrew after two vaccinations in the 1st course due to the rapid tumor progression. All the patients enrolled in this protocol were judged to have progressive disease (PD) after the first course of treatment and at final evaluation.

### Immunohistochemical analysis of vitiligo

**Figure 2:**
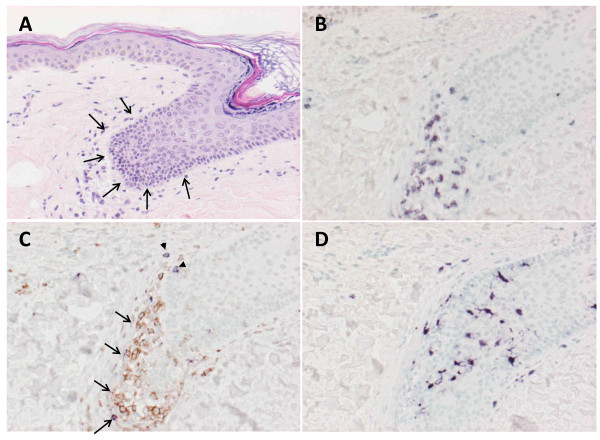
**Immunohistochemical analysis of vitiligo**. Biopsy specimens of the area with vitiligo in P2 were stained with H&E to identify infiltrating cells. This focus was a match for vitiligo. To characterize the nature of infiltrating lymphocytes and DCs, biopsy specimens were stained for cell surface markers that were antibodies against CD3, CD4, CD8 and S100. Marked lymphocytes infiltrated into epidermis with depigmentation of biopsy specimen stained with H&E (3A: arrow head). Immunohistochemical analysis revealed that CD3^+ ^T-cells infiltrated at the same sites (3B) and composed of CD4^+ ^T-cells (3C: arrow, brown) and CD8^+ ^T-cells (3C: arrow head, purple). Interestingly, DCs stained with S100 also accumulated at the same sites (3D).

As described in "***Adverse events***", two patients were found to have vitiligo throughout the body. These adverse events might be associated with the vaccination, since gp100 is also expressed by the non-cancerous melanocytes. The Fig. 1A shows vitiligo at the posterior portion of the neck in P2, and Fig. 1B showed vitiligo at the anterior tibial portion of the left leg in P3. The serial sections were made from tissue samples taken from the vitiligo of P2 and examined with H&E and immuno-histochemical staining (Fig. 2). The infiltration of CD3^+ ^T-cells was observed at the epidermis with de-pigmentation (Fig. 2A and 2B). These infiltrating T-cells mainly consisted of CD4^+ ^T-cells rather than CD8^+ ^T-cells (Fig. 2C). Interestingly, the S100-positive cells, compatible with a dendritic cell phenotype, accumulated in the same area (Fig. 2D). These findings suggest that the vitiligo is associated with the immunological responses promoted by the vaccination against gp100.

### Immunological monitoring for peptide specific T-cell responses in enrolled patients

**Figure 3:**
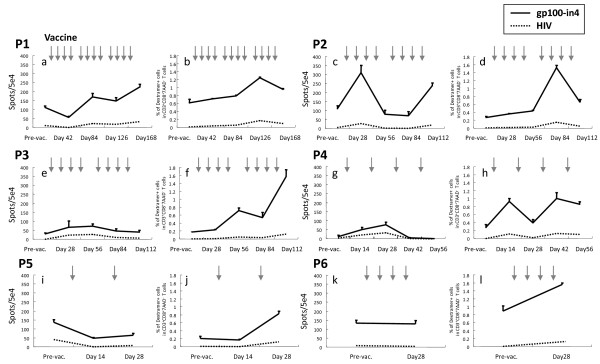
**Immunological monitoring for peptide specific T-cell responses in melanoma patients using ELISPOT and MHC-Dextramer assay **ELISPOT and the MHC-Dextramer assay are shown in the left (a, e, i, c, g and k) and right (b, f, j, d, h and l) side of each panel. The arrow indicates the timing of each vaccine injection. In ELISPOT assay, to evaluate the characteristics of peptide-specific CTLs, PBMCs of the patients were stimulated *in vitro *with gp100-in4, HIV or CMV peptide (data not shown) and examined for their ability to produce IFN-γ. The MHC-Dextramer assay was performed on blood samples identical to the ones used for ELISPOT assay to identify CD8^+ ^T-cells recognizing the epitope peptide used. For flow cytometric analysis, PBMCs, which were stimulated *in vitro*, were stained with the MHC-Dextramer for 20 min in the dark at room temperature, followed by staining with FITC-conjugated anti-CD8 mAb and APC-conjugated anti-CD3 mAb and 7AAD (Beckton Dickinson Biosciences) at 4°C for 30 min. Flow cytometric analysis was performed using FACSCalibur and CellQuest software (BD Biosciences).

As described in **Methods**, PBMCs obtained from the enrolled patients were examined with ELISPOT and MHC-Dextramer assay after the short-term culture. The results of the ELISPOT and the MHC-Dextramer assay are shown in the left and light panel for each patient in Fig. 3, respectively. In ELISPOT assay (Table 3), the responses of the patients were evaluated as ++ (strongly positive) if the numbers of IFN-γ positive spots after the vaccinations increased more than two fold compared with the numbers at pre-vaccination. If the increments were in between one to two fold, they were evaluated as + (marginally positive). These assays were performed at least three times to confirm reproducibility. To evaluate the characteristics of the peptide-specific CTLs, PBMCs of the patients were stimulated *in vitro *with either the gp100-in4, HIV or CMV peptide and examined for their ability to produce IFN-γ. IFN-γ-producing cells were induced with the gp100-in4 stimulation on the PBMCs taken from the patients (P1, P2, P3, and P4) after the vaccination. The PBMCs of P1 showed an incremental increase in the frequency of induced IFN-γ-producing cells in association with the number of the vaccinations. In P2, a significant increase of the frequency of induction of IFN-γ-producing cells was observed during the first course of the treatment. However, the frequency decreased soon after the first course of vaccination, but increased again with the second course of vaccination. In P3 and P4, IFN-γ-producing cells with significant specific reactivity to gp100-in4 were detected post vaccination, but at low levels. In contrast, IFN-γ-producing cells were not identified in the PBMCs taken from patients P5 and P6 even after vaccinations. P5 was withdrawn from the protocol due to the disease progression during the first course of the treatment.

**Table 3:** The results of immunological monitoring

Patients	Times of vaccination (Course)	Follow-up	ELISPOT assay (IFN-γ)	Dextramer assay (The frequency of peptide specific CTLs)
P1	12 (3)	13 months	++	+
P2	8 (2)	8 months	++	++
P3	8 (2)	7 months	+	++
P4	4 (1)	2 months	++	+
P5	2 (1)	1 month	-	++
P6	4 (1)	4 months	-	+

**The characteristics of patients exhibiting objective immunological responses**. In ELISPOT assay, the responses of the patients were evaluated as ++ (strongly positive), if the numbers of IFN-γ positive spots after the vaccinations increased more than two fold when compared with the numbers at pre-vaccination. If the increase was in between one to two fold, they were evaluated as + (marginally positive). These assays were performed at least three times to confirm reproducibility.

The MHC-Dextramer assay was performed on blood samples identical to the ones used for ELISPOT assay to identify the CD8-positive T-cells recognizing the epitope peptide. The results of the MHC-Dextramer assay are shown in Fig. 3. The frequency of CTLs specific for the gp100-in4 peptide was elevated two to eight times when compared with the pre-vaccination levels. In addition, the frequencies of the cells positive for both CD8^+ ^and HLA-A*2402/gp100-in4 Dextramer were higher than that at pre-vaccination. If the increases were more than 2-fold, they were evaluated as ++ (Table 3). All patients were judged to have positive responses.

### Establishment of gp100-specific CTL clones

**Figure 4:**
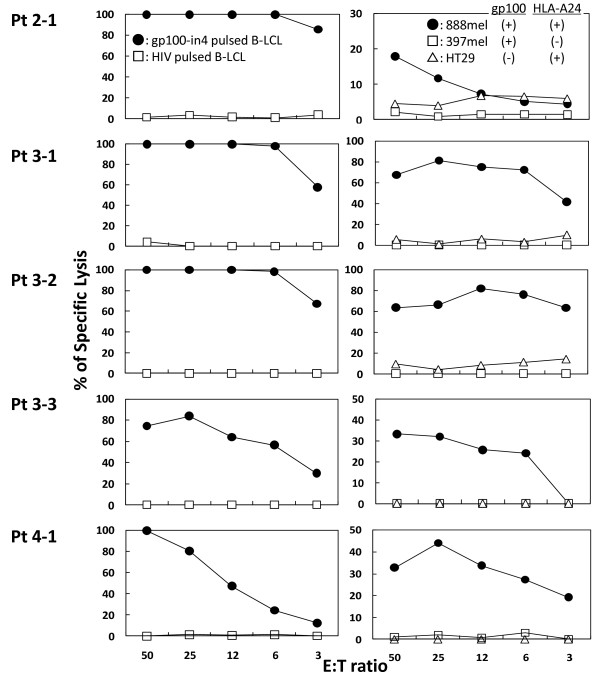
**Establishment of gp100-specific CTL clones**. The gp100-specific CTL clones were established in P2 and P3. Peptide-stimulated PBMCs with the ability to produce IFN-γ were expanded in the presence of feeder cells to establish gp100-specific CTL clones. Six clones were established (1 clone for P2, 3 clones for P3, and 2 clones for P4). A standard 4 h ^51^Cr-release assay was employed to confirm the anti-tumor response of these six clones. Representative results of the clones established from mP3 (P3-2, P3-3) are shown in the figure. All CTL clones were able to lyse A24-LCLs target cells pulsed with gp100-in4 (●), but not those pulsed with HLA-A*2402-resticted HIV peptide (□) in the left lane. They also were able to lyse 888 mel naturally expressing gp100 [gp100 (+), HLA-A24(+)]: (●) but not to lyse 397 mel [gp100 (+), HLA-A24(-)]: (□) and HT29 [gp100 (-), HLA-A24(+)]: (□).

CTL clones were generated from the patients' PBMCs as described above. One CTL clone from P2, three CTL clones from P3, and one clone from P4 were established from the PBMCs taken after the vaccination. No CTL clone was successfully established from the PBMCs taken before the vaccinations. A standard 4 h ^51^Cr-release assay was employed to confirm the cytotoxicity of these four CTL clones. Representative results of the cytotoxicity assay of all the CTL clones established from patient P2, P3 and P4 (P2-1, P3-1, P3-2, P3-3 and P4-1) are shown in Fig. 4. All the CTL clones were able to lyse A24-LCL target cells pulsed with gp100-in4 peptide, but not those pulsed with HIV peptide. These CTL clones also were able to lyse 888 mel, which naturally express gp100 [gp100 (+), HLA-A24 (+)], but were unable to lyse 397 mel [gp100 (+), HLA-A24 (-)] or HT29 [gp100 (-), HLA-A24 (+)]. These data provide evidence that the clones were gp100-specific. Similar results were obtained with other CTL clones from patient P2 (1 clone), P3 (2 clones), and P4 (1 clone). All the CTL clones were tested for the expression of the T-cell receptors binding to the HLA/peptide complex using the HLA-A*2402/gp100-in4 Dextramer. Similar results also were obtained from gp100-specific CTL clones established from P3.

### Inhibition of the specific cytotoxic reactivity with HLA-class I and CD8 monoclonal antibodies (mAbs)

**Figure 5:**
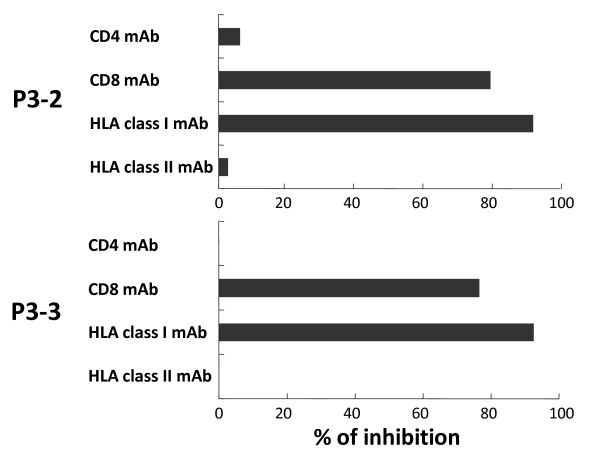
**Inhibition of the specific reactivity by mAb of HLA-class I and CD8**. The specific reactivity of CD8^+ ^T-cells was inhibited by mAb of HLA-class I and CD8. ^51^Cr-labeled 888 mel [gp100 (+), HLA-A24 (+)] pre-incubated with anti-class I MAb, anti-class II mAb, or a gp100-specific CTL clones (P3-2 or P3-3) were incubated with anti-CD4 mAb or anti-CD8 mAb for 1 hour at 4°C. After this time, effectors and target cells were mixed at an E/T ratio of 30 and cytotoxicity was determined after 4 hours incubation at 37°C. The cytotoxicity of the CTL clone against 888 mel was significantly reduced by the anti-class I and anti-CD8 mAbs.

To determine the involvement of HLA molecules and T-cell receptors in the recognition of antigen by the gp100-in4-reactive CTL clones, the ability of anti-class I mAb, anti-class II mAb, anti-CD4 mAb, and anti-CD8 mAb to inhibit the cytolytic activity of P3-2 and P3-3 established from gp100-in4-stimulated PBMCs of P3 was examined. The cytotoxicity of the CTL clones against 888 mel was significantly reduced with anti-class I mAb and anti-CD8 mAb. These results suggest that the CTL clones recognize the gp100-derived epitopes in an HLA class I-restricted manner (Fig. 5).

## Discussion

A phase I clinical trial was performed using an HLA-A*2402-restricted epitope-peptide derived from gp100 to examine its safety as a primary endpoint and clinical and immunological responses as secondary endpoints. Six patients with stage IV melanoma were immunized with a vaccine consisting of the epitope peptide emulsified with IFA.

In two patients, grade III adverse events (hematological and non-hematological) were observed. These events were examined in detail by the members of the IRB, independent of the study group, and judged not to be related to the vaccination, but to the progress of the disease. Some treatment-related adverse events were observed, but none were judged to be greater than Grade I. Thus, this treatment appears to be tolerated by this type of patients.

No objective anti-tumor effects, defined by RECIST criteria, were observed in any of the enrolled patients in the present study. Despite the fact that no significant therapeutic effects were obtained with this treatment, gp100-in4-specific T cell responses were observed in the PBMCs taken from some of the enrolled patients post-vaccination. In P1, P2, P3 and P4, an increase in the frequency of IFN-γ-producing cells was detected with the peptide-specific ELISPOT assay. With the MHC-Dextramer assay, which can detect the T-cell receptor capable of binding specifically to the gp100-in4 peptide presented on a particular MHC molecule, an increase in frequency of T-cells with the gp-100-specific T-cell receptor was observed in all patients after the initiation of vaccination. These results suggest that the vaccination with gp100-derived peptide can frequently induce peptide-specific CTLs in the peripheral blood, even in the patients with advanced melanoma treated with multiple modalities. Thus, this peptide could be used as an antigen to initiate the immune response against certain tumors, at least in the peripheral blood, similar to studies performed with other epitope peptides [33-39].

Interestingly, P2 and P3 were found to have new vitiligo, which appears to be correlated to the anti-tumor immune responses [40], after the initiation of the treatment. Although the events were recorded as adverse events in this protocol, this observation might be associated with the immune responses observed against the gp100 peptide in peripheral blood. To address this question, the skin tissue specimens with vitiligo were taken from the P2 after the 1st course of the vaccination with written consent. Morphological and immuno-histochemical examination of the specimen showed that there was an infiltration of inflammatory cells at the epidermis corresponding to the vitiligo. The infiltrating cells were found to be CD3^+ ^T-cells, which mainly consisted of CD4^+ ^T-cells rather than CD8^+ ^T-cells. In addition, there were numerous cells compatible to dendritic cells in morphology and positive for S100 staining. These findings might suggest that the vitiligo might have emerged as a consequence of the attack by the CTLs specific to gp-100 [41]. The dendritic cells might have accumulated to the site of immune response, engulfed resulting damaged cells, and induced the promotion of CD4^+ ^T-cells, which cannot recognize class I restricted peptide, but can recognize the melanocyte antigens presented on MHC-class II [42]. These observations on vitiligo also may suggest that the CTLs detected with the immune monitoring in the peripheral blood might have functional cytotoxic activity [43-45].

It is important to note that no clinical tumor responses were noted even in the patients with vitiligo. These results suggest that tumor cells were not efficiently attached by the antigen-specific T-cells successfully induced with the vaccination through the multiple mechanisms suggested elsewhere [46-51]. In this regard, the discrepancies between the results of ELISPOT assay and the MHC Dextramer assay in some patients (P5 and P6) suggest the existence of the mechanisms to suppress the immune functions. In these patients, the dextramer-positive CTLs were observed, but the ELISPOT assays showed negative results. It might mean that CTLs with T-cell receptors recognizing the epitope peptide might not be functionally active in these patients. Although we have examined the frequency of regulatory T-cells in CD4-positive cells of PBLs obtained from all the enrolled patients, no significant tendencies were found (data not shown). Further examinations, especially on the tumor micro-environment, might yield more definitive insights. In order to overcome these emerging obstacles, multiple strategies including the co-administration of high dose IL-2 and anti-CTLA-4 antibodies with vaccine have been proposed for the clinical studies [52,53]. Approaches to recruit CTLs to the tumor site and to facilitate the CTL functions at the tumor site would need to be developed to increase the therapeutic potency of the cancer vaccine.

## Conclusions

The peptide vaccine consisting of HLA-A*2402-restricted epitope peptide derived from gp100 and IFA was safely administered to the stage IV melanoma patients in this phase I trial. Although no therapeutic effects of this treatment were observed in the enrolled patients, immunologic monitoring of the treated patients clearly showed that this vaccine is capable of initiating immune responses against the melanoma. Thus the further development of this agent to be used as an immunogenic antigen in vaccine related therapies against melanoma is warranted.

## Abbreviations

ALT: alanine aminotransferase; AST: aspartate aminotransferase; APC: allo-phycocyanin; BUN: blood urea nitrogen; CMV: cytomegalovirus; CT: computed tomography; cytomegalovirus; ELISPOT: enzyme-linked immunospot; FITC: fluorescein isothiocyanate; H&E: Hematoxylin and eosin; HIV: human immunodeficiency virus; IL: interleukin; mAb: monoclonal antibody; MRI: magnetic resonance imaging; PE: phycoerythrin; WBC: white blood cell

## Competing interests

The authors declare that they have no competing interests.

## Authors' contributions

TB and MSM conceived and coordinated the study, carried out immunological assay and data analysis. MSM wrote the draft of the manuscript. HT participated in the design of the study, and carried out the clinical research. AK, AI, NO, YI participated in the conduct and data management of the clinical protocol. YK provided information regarding the gp-100 peptide and assisted in the interpretation of immunological assays. HT provided general supervision and helped to draft and edited the manuscript. All authors read and approved the final manuscript.
